# A population-base survey on knowledge, attitude and awareness of the general public on antibiotic use and resistance

**DOI:** 10.1186/s13756-020-00768-9

**Published:** 2020-07-11

**Authors:** Clement Yaw Effah, Adwoa Nyantakyiwaa Amoah, Hong Liu, Clement Agboyibor, Lijun Miao, Jing Wang, Yongjun Wu

**Affiliations:** 1 College of Public Health, Zhengzhou University, Zhengzhou, 450001 China; 2 Department of Respiratory, the First Affiliated Hospital of Zhengzhou University, Zhengzhou, 450052 China; 3 School of Pharmaceutical Sciences, Zhengzhou University, Zhengzhou, 450001 China

**Keywords:** Antibiotic resistance, Antibiotic usage, Public knowledge, Attitude

## Abstract

**Objectives:**

This study was designed to assess the awareness and knowledge of antibiotic usage and antibiotic resistance among the general public in the Cape Coast metropolis of Ghana. It also tries to decipher whether the level of education and the professional status of an individual has a positive association with the level of knowledge on antibiotic resistance.

**Methods:**

A population-base survey involving members of the public was conducted from August to November 2019. A structured questionnaire was developed to collect data from 632 respondents. Data were analyzed through SPSS v.21 using Chi square statistics and multivariate regression. Differences in knowledge were evaluated using ANOVA and the assumption of equal variance was tested with Levene statistics.

**Results:**

The response rate was 74.3%. Lower educational status group had a greater knowledge level (39.7%) on antibiotic resistance. Despite the high score, the lowest educational status group, (*M* = 1.82, *SD* = 0.769), middle educational status group (*M* = 1.98, *SD* = 0.748), and the high educational status group (*M* = 1.88, *SD* = 0.773) were not significantly different from each other with regard to their general knowledge level on antibiotic resistance (*P* < 0.05). The study revealed that, working in the healthcare sector is a major contributor to the level of knowledge on antibiotic resistance.

**Conclusion:**

Given the scale of the issue on antibiotic resistance and the fact that attempts to resolve it will involve efforts on the part of all, it is important that the public is aware of the importance of the issue of antibiotic resistance, its implications and what they can do to address it. The level of knowledge among respondents with lower educational status should be enough evidence to introduce more educational campaigns on antibiotic resistance.

## Introduction

Antimicrobial resistance is a serious and ever increasing public health threat [1, 2]. Antibiotic resistance poses enormous challenges including, longer hospital stays, higher mortality rate [3], a great economic burden and intangible costs [3, 4]. Common medical routines and techniques in clinical medicine are becoming nearly impossible making the treatment of some common infections very difficult. This problem can be partly due to the high risk associated with antibiotic usage [5]. In one of the general meetings of the World Health Assembly in 2015, an action plan was drafted to help solve the increasing problem of antimicrobial resistance. In this action plan, one objective was to enhance public knowledge on antimicrobial resistance through effective communication, education and training. Throughout most educative campaigns, the information provided is often characterized by a framing technical and informative message. For example, during public health campaigns, the messages used is usually focused on the ways of encouraging responsible antibiotic use and also, these messages include information on why misuse of antibiotics and resistance to antibiotics are critical public health issues [6]. Although several factors play a contributing role in antibiotic resistance, inappropriate use of antibiotics has been identified as the main cause [7, 8]. Other causes may include the use of antibiotics in the food production chain and also within the animal production sector.

In a study by Costelloe et al. and Morgan et al. [9, 10], the inappropriate use of antibiotics were directly correlated with antibiotic resistance of which healthcare workers and patience were considered to play a crucial role. In the case of healthcare professionals, it is most likely due to inappropriate prescription while in patients, it is most likely due to overuse, not taking the full course of treatment through self-medication. Also, sharing medication with other people, keeping part of a treatment course for another occasion, acquisition of un-prescribed antibiotics from pharmacies are all factors that lead to antibiotic resistance [7, 11]. Therefore, in order to contribute in solving the problem of antibiotic resistance, one must ensure the appropriate use of these drugs [5, 12].

Ghana as a developing country is also faced with this global crisis of antibiotic resistance and as a result, there are national plans (Ghana National Action Plan for Antimicrobial Use and Resistance, 2017–2021) and strategies on antimicrobial stewardship to help tackle this problem. Despite this problem, there is still no known study addressing the level of knowledge and awareness on antibiotic resistance in the Cape Coast Metropolis and the entire country as a whole. I would highlight that, understanding public knowledge on antibiotics, is crucial in identifying the different attitudes of people which could subsequently help shape campaigns and policies addressing this problem [13, 14]. Knowledge about the risk associated with the use and misuse of antibiotic is the key to dealing with antibiotic resistance. But there is limited data on public knowledge and awareness about antibiotic use and resistance in Ghana. In view of this, this study was designed to assess the awareness and knowledge on antibiotic usage and antibiotic resistance among the general public in the Cape Coast Metropolitan Assembly of Ghana.

## Methods

### Participants and study design

A cross-sectional survey design was used for this study. Respondents were reached through a formal electronic and paper invitation requesting them to participate in this survey. For those participants that were contacted electronically, their emails were obtained through an earlier exercise that was carried out within the Municipality. Upon accepting the invitation, the electronic questionnaire was sent to them for completion. Also, some respondents that had no access to internet or respondents that we did not have their emails were recruited via street-intercepts, home visits and through smaller local associations. Convenient, purposive and proportionate stratified random sampling were employed as sampling techniques for this study. Convenient sampling was employed for those whom we had their email addresses. The purposive sampling technique was adopted in other to ensure all participants who took part in the study fell within the age of ≥18 years. Additionally, the population (communities) was grouped into strata of four (4) and random selection was done so that each member of the population had an equal chance of being included in the study. The respondents were selected based on age (≥18 years) and nationality (Ghanaians). The respondents were then grouped according to their educational level i.e. low (≤Junior high), middle (Senior high school or its equivalent) and high (University or its equivalent). The professions of the respondents were initially blinded to the researcher but this information was elicited through the questionnaire and was then used to group the general public into health workers and non-health workers. About 99.5% of respondents were based in the coastal part of Ghana, Cape Coast to be precise.

### Questionnaire/survey instrument

The questionnaire (Supplementary File) was structured to facilitate self-administration by the public. The questionnaire was distributed within a four-month period from August to November 2019. The questionnaire was designed to assess individuals’ knowledge on antibiotics and antibiotic resistance, and deliberate behaviors by respondents regarding the prudent use of antibiotics. In total, the survey consisted of 55 questions, comprising 5-point Likert-type scales, multiple-choice and open questions, distributed over four sections. The first section focused on whether the respondent or any member of his/her family has taken antibiotics either in the past or is taking it at the moment. Also data on reasons for taking those antibiotics were elicited through this section. Section two focused on the accessibility of antimicrobials by the respondents. In the third section, we focused on assessing the level of knowledge on the use, the side effects and antibiotic resistance. With 10 questions of ‘true–false-don’t-know items, the levels of knowledge on antibiotic resistance were measured. Questions on antibiotic use and antimicrobial resistance were retrieved from the Eurobarometer survey 79.4, [15, 16]. Section four also assessed respondents’ experiences, respondent-doctor relationship and infection prevention.

Gender, age, educational level, and profession were included as statistical controls in our regression tables. For gender, females were the reference category, whereas for age, year group 18–21 was used as the reference. Lower education level and health workers were the reference categories for educational level and profession respectively. Educational level was measured with three categories: low, middle, high. The history of antibiotic use within the last 12 months was measured with four categories: never, once, 2–5 times and more than 5 times and a dummy category for those who could not recall. Although the data were not collected anonymously, we guaranteed complete confidentiality to each respondent. Regression analysis was performed according to respondents’ attitude and general awareness on antibiotic resistance per knowledge level.

### Data analysis

All responses were coded and analyzed using SPSS version 21. The demographic data of respondents were analyzed using descriptive statistics and results were presented as frequencies and percentages. The researchers employed simple statistical analyses (Chi square test) to determine whether a relationship existed between responses to questions on antibiotic use and misuse, perception of respondents on the role of health professionals in antibiotic use and antibiotic resistance, behavior and respondents’ demographics. A regression analysis on various demographics, was performed according to their level of knowledge on antibiotic use and antibiotic resistance. The differences in knowledge on antibiotic resistance among the various groups (educational level) were tested using the analysis of variance (ANOVA) to determine if the data met the assumption of equal variance (Levene statistics). Three scores were created to measure the level of knowledge on antibiotic use and antibiotic resistance based on a 10-level structured question. Each score was defined as the proportion of questions for which the answers were correct. These three scores ranged from 0 to 100%. For each respondent, the total of the correct answers on the knowledge items was computed and the scores were summed up. Based on their scores, respondents were ranked into groups as ‘low knowledgeable’ (having a maximum of four correct answers), ‘moderate knowledgeable’ (having up to seven correct answers) and the third group, as ‘highest knowledgeable’ (getting more than seven correct answers).

## Results

### General characteristics of respondents

A total of 1000 respondents were initially invited to participate in this survey. Out of these, 851 agreed to the invitation. Six-hundred and thirty-two (632) respondents out of the 851 filled and returned the questionnaires representing a response rate of 74.3%. Among them, 323 (51%) were males whiles 309 (49%) were females and they were wildly distributed within age groups 18-21 year (125, 20%), 22-25 years (257, 41%) and above 25 years (250, 40%; the oldest in the group was 60 years). The respondents were grouped based on their level of education with high educational status recording the highest proportion (359, 57%) followed by middle educational status (142, 22%) and low educational status (131, 21%) in that order. Four-hundred and twenty-four (424) of the respondents were non-health workers representing 67% of the total respondents.

### Knowledge on antibiotics, antibiotic use and accessibility, side effects and antibiotic resistance

From Table 1, it can be seen that a fairly average number of the respondents have a general idea about the common types of antibiotics available. This knowledge was significantly different across the various age groups, gender, and profession but non-significant base on their various educational levels. Furthermore, almost half of the respondents had ample knowledge on the fact that antibiotics are ineffective against the cold with statistical significance existing between the various age groups and profession (*P* < 0.05). Although the respondents had some knowledge on whether antibiotics are effective against common cold, barely few people within the different educational status, disagreed to the statement that, leftover antibiotics can be saved for personal future use; i.e. 60% (79/131) for low educational status, 33% (47/142) for middle educational status and 53% (191/359) for respondents within the higher educational status category. Responses to the various questions; 1. the body can fight mild infections on its own without antibiotics (agree) and 2. whether it is good to acquire antibiotics from relatives, without having to be examined by a doctor (disagree) were not significantly different (*P* < 0.05) among the various categories of educational status.

**Table 1:** Misconceptions of respondents on the use and misuse of antibiotics

Statements	Gender % (n) % = n/N		Age (Years) % (n) % = n/N			Educational Level % (n) % = n/N			Profession % (n) % = n/N	
	Male *N* = 323	Female *N* = 309	18–21 *N* = 125	22–25 *N* = 257	> 25 *N* = 250	Low *N* = 131	Middle *N* = 142	High *N* = 359	Health Worker *N* = 208	Non-health worker *N* = 424
Chloramphenicol, Kavepenin, Ampicillin are types of antibiotics (2/3 or 3/3 correct choices)	57 (184)	77 (246)	78 (98)	63 (162)	68 (170)	66 (86)	67 (99)	68 (245)	59 (123)	72 (307)
*X*^*2*^	37.297			24.508		8.904			45.005	
*P*	< 0.05			< 0.05		0.064			< 0.05	
Antibiotics make one recover faster when having a cold (Disagree)	16 (50)	13 (40)	12 (15)	16 (40)	14 (35)	15 (20)	9 (13)	16 (57)	27 (56)	8 (34)
*X*^*2*^	8.294		17.056			8.558			64.431	
*P*	0.081		< 0.05			0.381			< 0.05	
The body can usually fight mild infections on its own without antibiotics (agree)	62 (200)	55 (169)	54 (68)	48 (122)	72 (179)	72 (94)	56 (79)	55 (196)	79 (165)	48 (204)
*X*^*2*^	5.372		53.716			31.913			66.340	
*P*	0.372		< 0.05			< 0.05			< 0.05	
Leftover antibiotics can be saved for personal future use (disagree)	50 (160)	51 (157)	36 (45)	53 (137)	54 (135)	60 (79)	33 (47)	53 (191)	61 (126)	45 (191)
*X*^*2*^	3.281		26.682			39.550			15.149	
*P*	0.512		< 0.05			< 0.05			< 0.05	
It is good to acquire antibiotics from relatives, without having to be examined by a doctor (disagree)	58 (186)	56 (172)	52 (65)	59 (151)	57 (142)	63 (82)	47 (66)	59 (210)	67 (139)	52 (219)
*X*^*2*^	15.551		18.533			20.954			41.316	
*P*	< 0.05		< 0.05			< 0.05			< 0.05	

The number of correct answers on the knowledge items were determined for each respondent and the scores were computed and summed up. The first group, the ‘low knowledgeable’ had a maximum of four correct answers (*N* = 155, 25%), the second group, the ‘moderate knowledgeable’ had up to seven answers correct (*N* = 253, 40%) and the third, the ‘highest knowledgeable’ had more than seven answers correct (*N* = 224, 35%). Comparing the knowledge (high knowledge) on antibiotic use and antibiotic resistance based on the educational status, Lower educational status group recorded 39.7% (52/131) followed by the higher educational status group 36.5% (131/359) and Medium educational status 28.9% (41/142) (Table 2). A clear majority (480) representing 75.9%, of the total 632 respondents, had knowledge on bacteria’s ability to become resistant to antibiotics. Also, only a few knew that antibiotic resistant bacteria is transmissible among persons (34.8%, 220), between animals and humans (33.7%, 213) and that the human body itself cannot become resistant to antibiotics (14.4%, 91).

**Table 2:** Level of knowledge on antibiotics and antibiotic resistance among the respondents

(1) Antibiotics often cause side effects such as diarrhoea (True)								
(2) Antibiotics cause negative effects on the body’s own bacterial flora (True)								
(3) If one feels better after only partially completing an antibiotic course, one can terminate the therapy immediately (False)								
(4) Bacteria can become resistant to antibiotics (True)								
(5) The more antibiotics we use in society, the higher is the risk that resistance develops and spreads (True)								
(6) People can become resistant to antibiotics (False)								
(7) Antibiotic use for animals can reduce the possibility of effective antibiotic treatment for humans (True)								
(8) Resistance can spread from animals to humans (True)								
(9) Resistance can spread from person to person (True)								
(10) People travelling outside their home country risk bringing resistance upon return to their country (True)								
Results								
Statement	Gender		Age			Educational level		
	Male *N* = 323	Female *N* = 309	18–21 *N* = 125	22–25 *N* = 257	> 25 *N* = 250	Low *N* = 131	Medium *N* = 142	High *N* = 359
	Correct	Correct	Correct	Correct	Correct	Correct	Correct	Correct
(1)	46	31	26	32	52	51	32	37
(2)	57	49	42	48	64	70	39	52
(3)	68	68	68	59	77	73	61	69
(4)	78	74	63	73	85	79	73	76
(5)	72	57	51	64	72	73	61	63
(6)	15	14	16	17	11	13	14	15
(7)	35	22	19	28	33	31	18	31
(8)	44	23	25	35	37	39	25	35
(9)	39	30	33	36	34	36	27	38
(10)	42	31	33	39	36	43	31	36

Correct answer category is given after the statement (false/true). Scores are in percentages

From Table 3, regression analysis was performed on the general awareness on antibiotic resistance per knowledge level. Using the ANOVA, we found that variances among the various educational status were equal as we found a non-significant Levene statistic of 1.8999 (*P* = 0.151). The lowest educational status group, (*M* = 1.82, *SD* = 0.769), middle educational status group (*M* = 1.98, *SD* = 0.748), and the high educational status group (*M* = 1.88, *SD* = 0.773) were not significantly different from each other with regard to their general knowledge level on antibiotic resistance (*P* < 0.05). The expectation that the profession of the respondents will have an effect on the level of knowledge on antibiotic resistance was tested with the multivariate regression analysis displayed in Table 3. In this study, a person’s profession was a major contributor to the level of knowledge on antibiotic resistance as the lowest knowledgeable group had (*B* = -1.724, *SE* = 0.289), middle knowledgeable group (*B* = -0.648, *SE* = 0.190) and then the highest knowledgeable group had (*B* = 1.716, *SE* = 0.194) at (*P* < 0.01) (Table 3).

**Table 3:** Regression estimates on the general awareness of antibiotic resistance per knowledge profiles

Variables	Low Knowledge		Middle Knowledge		High Knowledge	
	B	SE	B	SE	B	SE
**Gender**						
Male (female reference)	−0.928***	0.229	−0.062	0.189	0.836***	0.209
**Age** (18–21 reference)						
22–25	0.006	0.308	0.494	0.263	−0.644**	0.299
> 25	0.414	0.242	0.126	0.197	−0.540***	0.208
**Educational Level** (lower reference)						
Middle	−0.106	0.269	−0.055	0.219	0.157	0.231
High	−0.046	0.254	0.239	0.216	−.0293	0.241
**Profession** (health workers reference)						
Non-health worker	−1.724***	0.289	−0.648***	0.190	1.716***	0.194
**Antibiotic use within last 12 months** (Never reference)						
Once	−0.310	0.379	0.887***	0.319	−0.847**	0.350
2–5 times	−0.420	0.343	0.827***	0.297	−0.693**	0.329
> 5 times	0.190	0.320	0.024	0.294	−0.265	0.318
**Anyone in your household taking antibiotics at the moment (Yes reference)**						
No	−0.203	0.315	0.230	0.265	−0.029	0.295
Don’t know	−0.349	0.233	−0.006	0.197	0.295	0.218
**Constant**	−0.246	0.745	−1.231	0.653	−0.413	0.738
**Cox & Snell R**^**2**^	0.144		0.062		0.190	
**Nagelkerke R**^**2**^	0.214		0.084		0.261	

*** *p* < .01; **p < .05

### Patient experiences, patient-doctor relationships and infection prevention

The attitude of respondents toward antibiotic use, infections and hygienic practices is the key to reducing antibiotic resistance. These were measured using Chi-square analysis and reported among the various variables. In totality, 39.7% (251/632) opined that, when they are challenged with infections, they wait and see, i.e. rest without any medication and later monitor if the infection goes away on its own. Also 62.2% (393/632) usually use hand hygiene to reduce the risk of spreading common infections. Most importantly, the majority of them (73.1%, 462/632) (Table 4) used doctor’s prescription to purchase antibiotics from pharmacies. From Table 5, some respondents across gender, age, profession and educational status reported that; (1) doctors don’t conduct a thorough examination regarding whether a patient is in need of antibiotics (2) doctor don’t take time to provide information on how antibiotics should be used and (3) pharmacy staffs do not take their time to inform them on how antibiotics should be used.

**Table 4:** General attitudes of respondents on acquisition, use and hygienic practices to reduce antibiotic resistance and its’ spread

Statements on antibiotic use and Misuse (correct responses)	Gender % (n) % = n/N		Age (Years) %(n) % = n/N			Educational Level % (n) % = n/N		
	Male *N* = 323	Female *N* = 309	18–21 *N* = 125	22–25 *N* = 257	> 25 *N* = 250	Low *N* = 131	Middle *N* = 142	High *N* = 359
If I get an infection, I often wait and see, i.e. rest and take it easy, and see if the infection goes away on its own (Yes)	34 (109)	46 (142)	41 (51)	39 (99)	40 (101)	52 (68)	35 (50)	37 (133)
*X*^*2*^	23.705		10.138			35.181		
*P*	< 0.05		0.256			< 0.05		
I usually use hand hygiene (hand washing or alcohol hand rub) to reduce the risk of spreading common infections (Yes)	64 (206)	61 (187)	76 (70)	62 (159)	67 (164)	66 (87)	69 (98)	60 (208)
*X*^*2*^	22.582		31.482			17.585		
*P*	< 0.05		< 0.05			0.062		
I always use doctor’s prescription to purchase antibiotics from pharmacies (Yes)	75 (241)	72 (221)	63 (79)	69 (177)	82 (206	84 (110)	65 (92)	72 (260)
*X*^*2*^	0.768		19.564			12.943		
*P*	0.381		< 0.05			< 0.05

**Table 5:** Perception of respondents on the role of health professionals in antibiotic use and antibiotic resistance

Statements	Gender % (n) % = n/N		Age (Years) % (n) % = n/N			Educational Level %(n) % = n/N		
	Male *N* = 323	Female *N* = 309	18–21 *N* = 125	22–25 *N* = 257	> 25 *N* = 250	Low *N* = 131	Middle *N* = 142	High *N* = 359
Doctors always conduct a thorough examination regarding whether a patient is in need of antibiotics or not (agree)	42 (134)	49 (152)	50 (63)	41 (104)	48 (119)	37 (49)	51 (72)	46 (165)
*X*^*2*^	6.364		22.346			27.750		
*P*	0.174		< 0.05			< 0.05		
Doctors prescribe antibiotics when a patient expects it (agree)	17 (55)	26 (81)	30 (38)	19 (48)	20 (50)	24 (31)	32 (45)	17 (60)
*X*^*2*^	10.899		26.817			35.166		
*P*	< 0.05		< 0.05			< 0.05		
When antibiotics are prescribed, the doctor takes time to provide information on how they should be used (No)	38 (123)	29 (89)	22 (27)	31 (79)	42 (106)	34 (45)	25 (36)	37 (131)
*X*^*2*^	6.098		17.702			5.711		
*P*	< 0.05		< 0.05			0.058		
Pharmacy staff take their time to inform me on how antibiotics should be used (No)	30 (97)	24 (74)	26 (33)	31 (79)	24 (59)	20 (26)	20 (28)	33 (117)
*X*^*2*^	2.961		3.307			12.895		
*P*	0.085		0.191			< 0.05		

## Discussions

The problem with antibiotic use and resistance has been a major challenge to the public health sectors. To overcome the problem of antibiotic resistance, the number of times antibiotics are taken within a specified time is crucial. The results of the survey on antibiotic use demonstrate how frequently antibiotics are taken, with a considerable majority of respondents confirming the consumption of antibiotics (2–5 times) within the last 12 months. This prevalence is highly relevant to public campaigns on antibiotic resistance—both because high levels of use contribute to the problem, and because it demonstrates just how many people it could impact in a short time frame if the antibiotics they are taking become increasingly ineffective.

Antibiotics and its resistance need to be given a much greater attention. From this study, approximately half of the respondents still believe that antibiotics can be saved for personal use and that, it is a good habit to acquire antibiotics from relatives without any thorough checkup by a physician. Various studies have confirmed that keeping leftover antibiotics results in poor compliance with antibiotic therapy [17], and this also contributes to antibiotic resistance [7, 17], however, the potential three-fold impact on antibiotic resistance has not yet been fully realized. Firstly, when one does not complete the full course of an antibiotic due to temporal recovery, surviving bacteria can proliferate which may lead to antibiotic resistance. According to the WHO, when a patient stops the intake of antibiotics too early, it favours the bacteria strains that have some natural intrinsic resistance and it is therefore advisable that patients always take the full course of antibiotics prescribed to them by a certified health professional. Secondly, leftover antibiotics may not be the right drug for new infections as various infections require various antibiotics. Thirdly, to treat any infection, one requires a sufficient quantum of drug for treatment duration. Therefore, if there is any leftover antibiotic, it may be insufficient for treatment duration [17]. Subsequently, the general public needs to be educated and made aware on the fact that, the effectiveness of an antibiotic can be preserved only when they are used with a valid prescription and also when the full course is completed [17, 18]. To minimize the problem of antibiotic resistance, healthcare workers can play a key role through communication. Appropriate usage of antibiotics by patients can be conveyed by healthcare providers through effective communication. Horne [19] reiterated this fact by stressing the importance of patient-healthcare provider interaction and communication. This interaction can help promote optimal adherence to antibiotic usage by patients. Yet results from this study revealed that, some respondents across gender, age, profession and educational status reported that; doctors and pharmacy staffs do not take time to communicate to them on how antibiotics should be used.

Furthermore, one contributor to misuse of antibiotics is the lack of understanding of which conditions can or cannot be treated with antibiotics and the lack of such knowledge can lead to the development of resistance. On average, only 14.2% (90/632) of the respondents across the various gender, age, educational status and profession, disagree to the statement that antibiotics make one recover faster when having a cold. This is because the respondents are not aware that common cold and flu are not caused by bacteria but viruses and does not need an antibiotic to treat it. About three quarters (75%) of respondents believed that, people can become resistant to antibiotics and that antibiotic resistant bacteria cannot spread from person to person. It should be noted that, antibiotic-resistant bacteria can spread from person to person, with the potential to affect anyone, of any age and in any country. The majority of respondents agreed to the fact that, the more antibiotics are used in a society, the higher are the risk that resistance develops and spreads.

Past and current public health campaigns on antimicrobial resistance have echoed a key assumption in the so-called ‘information deficit literature’ [20], that, the low levels of public acceptance on proper use of antibiotics are the reflection of low levels of awareness of antibiotic use and knowledge about the possibly disastrous effects of improper use. This study agrees to this in the sense that, there is still a great deficit in knowledge on the awareness on the dangers that comes with antibiotic misuse and its resistance. The current results are in agreement to a study by McNulty et al. [21], whose results suggested that there is a need to improve the status quo because there is still some minimal improvement in the knowledge level of the general public on antibiotic resistance. From the current study, it can be deduced that, employment in the heath sector had a positive significant effect on knowledge about antibiotic use and antibiotic resistance in the multivariable analysis. Healthcare professionals play an important role in tackling the problem of antibiotic resistant. Therefore, an up to date knowledge on the dangers of misuse and antibiotic resistance can help them educate their patients on the potential risks. Age, Educational level, and whether anyone in the respondent’s household is taking antibiotics at the moment had no association with their knowledge level on antibiotic resistance.

A lot of studies have proved that people with high level of education have much knowledge on antibiotic resistance compare to people with low level of education [22, 23]. This study however, is in contradiction with the above findings which shows that education on antibiotic resistance have gone down well on people with low educational background. On a second enquiry into the possible reason which may have accounting for the observed results in this municipality, it was realized that some level of education has been embarked on for the past two (2) years where people (especially people with low level of education) are educated on the use and misuse of antibiotic. The tremendous level of knowledge among the lower educational status group disagrees with studies conducted in Italy, United Kingdom, Hong Kong, Sweden and Poland which reported a lack of knowledge on antibiotic resistance among this group [21–27]. This great improvement may be as a result of the educational campaign embarked on by various associations (Ghana Science Association) and other organizations (Ghana Pharmaceutical association) in the Municipality. Although these educational campaigns were not the focus of this articles, it can be linked to the tremendous rise in the level of knowledge on antibiotic resistances by people with low educational status (no senior high school education). Eng et al. [28], concluded that people’s knowledge and attitude regarding antibiotic use and its resistance can be substantially improved through education which was realized in this study. This improved knowledge might be important efforts to reduce misconceptions and misguided expectations contributing to inappropriate antibiotic use. This study is also in agreement with Ayepola et al. [29] whose findings iterated the fact that lower level students had more knowledge on antibiotic resistance than their counterparts with higher level of education. This gives an indication that, people with high level of education are either saturated with knowledge or they do not pay much attention to educational campaigns on antibiotic resistance.

### Strengths and limitations

This study fills the paucity of information on knowledge on antibiotic use and resistance. Our findings provides a repository of data which will help shape campaigns and policies addressing this problem. The strength of this study is the fact that it employed a reasonably large number of respondents considering the fact that this is the first known population base study within the municipality. Also, respondents were sampled from the general population and not from the hospital setting as most studies do. Limitations of this study were; 1. A possible bias towards respondents who may have a fair knowledge on the subject matter and 2. By using closed-ended questions to assess the level of knowledge on antibiotic resistance, respondents may have selected the most favourable answer instead of using qualitative methods to revel misconceptions.

## Conclusions

Given the magnitude of the problem of antibiotic resistance and the fact that attempts to resolve it will involve efforts on the part of all, it is important that the public is aware of the importance of the issue of antibiotic resistance, its implications and what they can do to address it. The level of knowledge among respondents of the lower educational level is a positive direction which we can leverage on to deal with the problem of antibiotic resistance. Strategies that can be employed by healthcare workers to improve the communication between them and their patients on effective ways to use antibiotics should be a focal point by researchers and healthcare authorities.

## Supplementary information

**Additional file 1.** Survey Instrument on antibiotic resistance.

## Data Availability

The datasets used and/or analyzed during the current study are available from the corresponding author on reasonable request.
