# Supplementary material for: A population-base survey on knowledge, attitude and awareness of the general public on antibiotic use and resistance
Source: Antimicrob Resist Infect Control. 2020 Jul 11;9:105. doi: 10.1186/s13756-020-00768-9 (PMC7353772; doi:10.1186/s13756-020-00768-9)
Supplement: Supplementary file 1 — Additional file 1. Survey Instrument on antibiotic resistance. [file 13756_2020_768_MOESM1_ESM.docx]

**Survey Instrument on antibiotic resistance**

1. **Antibiotic consumption**

*Antibiotics are medications which are sometimes used to treat infections. There are several different types of antibiotics; penicillin is the most common.*

- 1. **Which of the following medications are antibiotics?** *Please tick one or more options.*
     - Paracetamol □ Chloramphenicol
     - Seloken □ Ampicillin
     - Kavepenin □ Alvedon
  2. **Have you ever taken antibiotics?** *Please tick only one option.*
     - Yes
     - No
     - Don’t know
  3. **How many times have you consumed antibiotics during the past 12 months?** *Please tick only one option.*
     - Never
     - Once
     - 2-5 times
     - More than 5 times
  4. **How many times has another adult in your household (over 18 years old) received antibiotics during the past 12 months?** *Please tick only one option.*
     - Never □ Don’t know
     - Once
     - 2-5 times
     - More than 5 times
     - There are no other adults in the household
  5. **How many children under the age of 18 live in your household on a regular basis?** *Please tick only one option.*
     - 0

□ 1

□ 2

- - - 3 or more

**1.6 How many times have children in your household received antibiotics during the past 12**

**months (in total)?** *Please tick only one option.*

□

□

□

□

Never

Once

2-5 times

- Don’t know

More than 5 times

**1.7 Is anyone in your household taking antibiotics at the moment?** *Please tick only one option.*

□

□

□

Yes, for what reason? Please specify the person’s age _ years

No

Don’t know

1. **Antibiotic accessibility**

*The following questions should be answered using the scale below. Use this scale to indicate to what extent you agree with the question/statement. Please tick only one option.*

|  | Totally agree | Don’t agree at all | Don’t know |
| --- | --- | --- | --- |
| 2.1 Leftover antibiotics can be saved for personal future use or to give to someone else. | □ | □ | □ |
| 2.2 Leftover antibiotics should be taken back to the pharmacy. | □ | □ | □ |
| 2.3 I think that it is good that one needs a prescription to acquire antibiotics from pharmacies in Ghana. | □ | □ | □ |
| 2.4 I think that it is good to be able to buy antibiotics online, without having to see a doctor. | □ | □ | □ |
| 2.5 I think that it is good to be able to acquire antibiotics from relatives or acquaintances, without having to be examined by a doctor. | □ | □ | □ |
| 2.6 It is good that one can buy antibiotics without a prescription in pharmacies within certain countries. | □ | □ | □ |

1. **Antibiotic use and its effects**

|  | Totally agree | Don’t agree at all | Don’t know |
| --- | --- | --- | --- |
| 3.1 Antibiotics make one recover faster when having a cold. | □ | □ | □ |
| 3.2 If one’s mucous becomes coloured when having a cold, one always need antibiotics to get rid of the cold. | □ | □ | □ |
| 3.3 The body can usually fight mild infections on its own without antibiotics. | □ | □ | □ |
| 3.4 A persistent cough (longer than one week) always needs to be treated with antibiotics to disappear. | □ | □ | □ |
| 3.5 It is appropriate to take antibiotics when having a sore throat and a common cold, otherwise one may suffer complications. | □ | □ | □ |
| 3.6 Is it appropriate for cancer patients to take antibiotics before immunotherapy? | □ | □ | □ |
| 3.7 A lung infection in a 3-6 year old child always needs to be treated with antibiotics. | □ | □ | □ |
| 3.8 Lower respiratory tract infections can heal themselves without antibiotics. | □ | □ | □ |
| 3.9 By taking antibiotics one can often avoid staying home from work. | □ | □ | □ |
| 3.10 Antibiotics are supposed to kill all bacteria in the body. | □ | □ | □ |

1. **Side effects and resistance**

|  | Yes | No | Don’t know |
| --- | --- | --- | --- |
| 4.1 Antibiotics often cause side effects such as diarrhoea. | □ | □ | □ |
| 4.2 Antibiotics cause negative effects on the body's own bacterial flora. | □ | □ | □ |
| 4.3 If one feels better after only partially completing an antibiotic course, one can terminate the therapy immediately. | □ | □ | □ |
| 4.4 Bacteria can become resistant to antibiotics. | □ | □ | □ |
| 4.5 The more antibiotics we use in society, the higher is the risk that resistance develops and spreads. | □ | □ | □ |
| 4.6 People can become resistant to antibiotics. | □ | □ | □ |

|  | Yes | No | Don’t know |
| --- | --- | --- | --- |
| 4.7 Antibiotic use for animals can reduce the possibility of effective antibiotic treatment for humans. | □ | □ | □ |
| 4.8 Resistance can spread from animals to humans. | □ | □ | □ |
| 4.9 Resistance can spread from person to person. | □ | □ | □ |
| 4.10 People travelling outside their home country risk bringing resistance upon return to their country. | □ | □ | □ |

|  | Totally agree | Don’t agree at all | Don’t know |
| --- | --- | --- | --- |
| 4.11 Today, antibiotic resistance is a big problem in  Ghana. | □ | □ | □ |
| 4.12 Today, antibiotic resistance is a big problem in the world. | □ | □ | □ |

1. **Patient experiences, patient-doctor relationships and infection prevention**

|  | Totally agree | Don’t agree at all | Don’t know |
| --- | --- | --- | --- |
| 5.1 Doctors always conduct a thorough examination regarding whether a patient is in need of antibiotics or not. | □ | □ | □ |
| 5.2 Doctors prescribe antibiotics when a patient expects it. | □ | □ | □ |

|  | Yes | No |  |
| --- | --- | --- | --- |
| 5.3a I have experienced antibiotic prescription for myself or my kin. | □ | □ |  |
|  |  |  |  |
| 5.3b When antibiotics are prescribed, the doctor takes time to provide information on how they should be used, in an understandable manner. | □ | □ |  |
| 5.4 I usually know how antibiotics should be taken, even if I was given information about their use. | □ | □ |  |
| 5.5a I have experience, as a patient or as kin, of acquiring prescribed antibiotics from a pharmacy. | □ | □ |  |
| 5.5b Pharmacy staff take their time to inform me on how antibiotics should be used. | □ | □ |  |

|  | Totally agree | Don’t agree at all | Don’t know |
| --- | --- | --- | --- |
| 5.6 I often know before I visit a doctor, whether I need antibiotics or not. | □ | □ | □ |
| 5.7 I am confident in a doctor’s decision if s/he does not prescribe antibiotics. | □ | □ | □ |
| 5.8 A doctor who does not prescribe antibiotics when the patient thinks that they are needed, is not as good a doctor. | □ | □ | □ |
| 5.9 I am confident in a doctor's decision if s/he prescribes antibiotics. | □ | □ | □ |
| 5.10 I usually know how infections should be treated. | □ | □ | □ |
| 5.11 If I get an infection, I often wait and see, i.e. rest and take it easy, and see if the infection goes away on its own. | □ | □ | □ |
| 5.12 Hand hygiene (hand washing or alcohol hand rub) reduces the risk of spreading common infections, such as influenza. | □ | □ | □ |

|  | Totally agree | Don’t agree at all | Don’t know |
| --- | --- | --- | --- |
| 5.13 I am confident in the work that Ghana healthcare in carrying out to minimise the development of resistance. | □ | □ | □ |
| 5.14 I am confident that pharmaceutical companies will be able to develop new medicines which will solve the problem of antibiotic resistance. | □ | □ | □ |

**6. Background information**

**Gender Age**

□Male □Female _________Years

**Level of Education**

□Junior High School (or equivalent) □Senior High School (or equivalent)

□University (or equivalent)

**Profession**

□Health care worker □Non-health care worker

**Country where you received most part of your education**

­­­­­­­­­­­**______________________________________________**
